# Biochemical and hematological findings and risk factors associated with kidney impairment in patients with COVID-19

**DOI:** 10.5937/jomb0-37343

**Published:** 2023-01-20

**Authors:** Yosra Mohammed Yaghmour, Shraim Ala'a Said, Abbas Manal Ahmad

**Affiliations:** 1 Al-Ahliyya Amman University, Faculty of Allied Medical Sciences, Department of Medical Laboratory Sciences, Amman, Jordan; 2 Al-Ahliyya Amman University, Pharmacological and Diagnostic Research Lab, Amman, Jordan

**Keywords:** COVID-19, CBC, CRP creatinine, kidney, urine analysis, KOVID-19, CBC, CRP kreatinin, bubreg, analiza urina

## Abstract

**Background:**

COVID-19 is a new pandemic that has infected millions of people worldwide and caused a high morbidity and mortality rate. COVID-19 may have a harmful effect on organs, especially the kidneys. Aims: The main aim of our research is to study the association between the severity of COVID-19 disease and biochemical parameters related to kidney function and to investigate certain risk factors of COVID-19-associated kidney disease.

**Methods:**

A total of 174 individuals, 121 COVID-19 positive and 53 COVID-19 negative, were enrolled in this study. The relation between COVID-19 infection, severity, kidney function test, and hematological indicators were examined.

**Results:**

The most prominent symptoms among COVID-19 were fever (95% ) and fatigue (92%). Regarding biochemical parameters, median creatinine, MPV, and CRP were significantly higher in COVID-19 patients, whereas median eGFR, Na+, WBC, MCH, MCHC, and eosinophil percentages were significantly lower in this group. Severely infected patients were observed to have higher urea, creatinine, neutrophils, and NLR. However, median sodium, eGFR, hemoglobin, hematocrit, RBC, lymphocytes, and platelet count were significantly lower in the severe group. Urine examination of the severe group showed a significantly lower specific gravity, while urine pH, protein, and glucose were significantly higher.

**Conclusions:**

Our analysis indicates that COVID-19 infection affects kidney function, mainly creatinine level, urea, eGFR, Na+ and urine protein. Additionally, comorbidities such as older age (>65), hypertension, taking medications, and CRP (>33.55 mg/L) are considered risk factors that are more likely to contribute to kidney impairment in COVID-19 positive patients.

## Abstract (sr)

**Uvod:**

KOVID-19 je nova pandemija koja je zarazila milione ljudi širom sveta i izazvala visoku stopu morbiditeta i mortaliteta. KOVID-19 može imati štetan uticaj na organe, posebno na bubrege. Ciljevi: Glavni cilj našeg istraživanja je da se prouci povezanost između težine bolesti KOVID-19 i biohemijskih parametara vezanih za funkciju bubrega i da se ispitaju određeni faktori rizika od bolesti bubrega povezanih sa KOVID-19.

**Metode:**

Ova studija je ukljucila ukupno 174 osobe, od kojih je 121 bila pozitivna na KOVID-19, a 53 sa negativnim nalazom. Ispitivana je veza između ozbiljnosti infekcije, testa funkcije bubrega i hematoloških pokazatelja.

**Rezultati:**

Najistaknutiji simptomi među pacijentima obolelim od KOVID-19 su bili povišena temperatura (95%) i umor (92%). Sto se tice biohemijskih parametara, srednji kreatinin, MPV i CRP su bili znacajno viši kod pacijenata sa KOVID-19, dok su medijani eGFR, Na+, VBC, MCH, MCHC i procenti eozinofila bili znacajno niži u ovoj grupi. Primećeno je da pacijenti sa teškom infekcijom imaju povišenu ureu, kreatinin, neutrofile i NLR. Međutim, medijani natrijuma, eGFR, hemoglobina, hematokrita, eritrocita, limfocita i broja trombocita su bili znacajno niži u grupi pacijenata sa težim oblikom oboljenja. Ispitivanje urina kod grupe sa težim oblikom bolesti je ukazalo na znacajno manju specificnu težinu, dok su pH urina, proteini i glukoza bili znacajno viši.

**Zaključak:**

Naša analiza ukazuje da KOVID-19 utiče na funkciju bubrega, uglavnom na nivo kreatinina, ureju, eGFR, Na+ i proteine u urinu. Pored toga, komorbiditeti kao što su starija životna dob (>65), hipertenzija, uzimanje lekova i CRP (>33,55 mg/L) se smatraju faktorima rizika koji će verovatno doprineti oštećenju bubrega kod pacijenata pozitivnih na KOVID-19

## Introduction

Severe acute respiratory syndrome coronavirus 2 (SARS-CoV-2) was first reported in Wuhan at the end of 2019 [1]. This virus triggered a worldwide pandemic of the disease known as COVID-19 [2]. The clinical manifestation of the infection is highly variable. Indeed, older people and people with comorbidities have a higher risk of developing severe symptoms and complications [3] [4]. The angiotensin-converting-enzyme 2 is the key functional receptor for SARS-CoV-2 infection. In kidneys, it is found in podocytes and primarily in the brush border of proximal tubular cells [5]. Therefore, kidneys might also be important target organs for SARS-CoV-2. Emerging data indicate that SARS-CoV-2 infection contributes to renal damage through direct virus cytotoxicity and indirectly through cytokine storm syndrome [5]. As reported, renal deterioration is associated with a 5.3-fold in-hospital risk of death in COVID-19 patients [6]. Therefore, early recognition and treatment of AKI may limit associated complications such as long-term chronic kidney disease (CKD) and the need for dialysis [7].

Additionally, pre-existing CKD has been associated with an enhanced risk of severe COVID-19 infection [8] [9]. Accordingly, with 10%–15% of the global population of CKD, Zhang and Zhang (2020) addressed the critical need to elucidate who is vulnerable to kidney injury, what clinical symptoms there are and how early diagnosis can be made, and eventually how patients can be tracked during follow-up [10]. In Jordan, the number of people treated in the Dialysis Units in 2018 was 5318 [11]. In fact, a high-rate prevalence of under-diagnosed CKD was reported among Jordanians, with its association with demographic and clinical factors [12]. The main aim of our research is to study the association between the severity of COVID-19 disease and some hematological and biochemical parameters related to kidney function and to investigate certain risk factors of COVID-19associated kidney disease.

## Materials and methods

### Participants

One hundred twenty-one COVID-19 positive Jordanian adults and fifty-three age, and sex-matched COVID-19 negative subjects were included in the study.

### Ethical consideration

The research ethics committee approved this study by the faculty of Allied medical sciences at Al-Ahliyya Amman University (ethical approval number: AA-4-3-21) and the Ministry of Health (ethical approval number: MOH/REC/2021/171).

### Laboratory tests

Samples were collected after signing a consent form and describing the study aims and procedure to the patients. Five milliliters of blood sample were obtained from each participant and collected into a plain tube. Urine samples were self-collected in a labeled sterile container. Patients filled in a questionnaire in Arabic, giving general information about their demographic data, date of infection, symptoms, smoking and alcohol consumption, chronic diseases, medications, vitamin and food supplements, and dietary and physical habits. RT-PCR of a nasopharyngeal swab confirmed COVID-19 infection. Beckman Coulter kit was used to determine the value of hs-CRP. Creatinine (Cr), urea, blood urea nitrogen (BUN), uric acid, potassium, sodium, and chloride were analyzed using a standard chemistry analyzer (Beckman Coulter, AU480, USA). Beckman Coulter DXH 500 autoanalyser was used for CBC analysis. Urine was processed and evaluated by Dipstick (Combur-test strips, Roche). The estimated glomerular filtration rate (eGFR) was calculated using the CKD-EPI Creatinine Equation (2021) from the National Kidney Foundation, 2019.

### Data analysis

SPSS version 22.0 was used. Categorical variables were summarized as frequencies and percentages; numerical variables were represented by the mean (±SD) if they were normally distributed or the median (interquartile range) if they were not normal. Normality was checked by both Kolmogorov-Smirnov and Shapiro-Wilk tests. The difference between the two groups concerning numerical variables was carried out using an independent t-test if the distributions of both groups were normal; otherwise, the Mann-Whitney U test was used. The difference between groups concerning categorical variables was carried out using the Chi-square test of independence or Fisher's exact test if the expected counts in the contingency table were less than 5 in more than 20% of the cells or if the sample sizes were small. Binary logistic regression was performed with creatinine level as the binary outcome. Each risk factor was entered in a univariate model as the explanatory variable, and the 95% confidence interval (CI) odds ratio was calculated. The significance level was 0.05 except for the difference in the levels of eGFR. Post hoc residual tests were Bonferroni corrected for multiple testing, and the significance level was set at 0.01 since eGFR has five levels.

## Results

### Participant profile

A total of 174 individuals participated in this study, including 90 males and 84 females. The demographic, biochemical, and hematological parameters of all participants are shown in Table 1. The median age of the whole sample was 50 years.

**Table 1:** Demographic, biochemical, and hematological parameters of all participants N: Number of subjects with available measurements, Min.: Minimum, Max.: Maximum, IQR: Interquartile range (25th–75th percentile).<br>The normal ranges were according to the following references ^*^Winter et al. [13], 2014, ^**^Wu et al. [14], 2019.

Variable	Normal range	N	Min.	Max.	Median	IQR
Age (years)	……..	174	18	95	50	31.8–64
Female gender	……..	84<br>(48.2%)				
BMI (kg/m^2^) *	18.5–24.9	116	18.9	48.98	26.62	24.6–30.8
Kidney function						
Creatinine (μmol/L)	Female 53.0–106.0<br>Male 61.8–114.9	174	35.3	389.8	76.9	61.8–106.0
BUN (mmol/L)	2.8–8.1	174	1.5	48.1	5.1	3.8–8.2
BUN/creatinine	0.79–1.58	174	6.7	63.9	17.7	13.5–22.9
Uric acid (mmol/L)	Female0.13–0.39<br>Male0.26–0.45	174	0.11	0.96	0.31	0.25–0.40
Potassium (mmol/L)	3.5–5.3	174	3.2	5.71	4.29	4.1–4.5
Sodium (mmol/L)	135–145	174	120	148	135.5	134–139
Chloride (mmol/L)	98–111	174	88	120	102	100–105
eGFR (mL/min/1.73 m^2^)	90–120	174	9.8	153.3	90.7	60.7–111.6
CBC						
Hb (g/L)	138–180	95	78	190	144	134–160
Hct (%)	42.0–54.0	95	24.7	55.7	43.9	40–48
RBC (10^12^/L)	4.06–5.63	95	2.6	6.66	5	4.4–5.5
MCV (fL)	76.0–94.0	95	48.1	108.9	89	86–93.1
MCH (pg)	27.0–33.0	95	16.1	36	29.7	28.3–31
MCHC (g/L)	320–360	95	186	358	334	327–339
RDW (%)	11.6–15.0	95	12.6	22.6	13.9	13.4–14.5
MPV (fL)	7.2–11.7	95	6.9	13	8.8	8.2–9.5
WBC (10^9^/L)	4.0–11.0	95	3.3	16.1	6.8	5.4–8.6
Neutrophils (%)	40–60	95	9	95	58	49–66
Lymphocytes (%)	20–40	95	2	76	32	25–40
Monocytes (%)	2–8	95	1	14	8	6–9
Eosinophils (%)	1–3	95	0	9	2	1–2
Basophils (%)	0–1	95	0	1	0	0–0
Platelets (10^9^/L)	150–450	95	100	522	244	209–301
Neutrophil/lymphocyte**	1–3	95	0.1	47	1.8	1.3–2.7
Urinalysis						
Urine specific gravity<br>Urine pH	1.005–1.030<br>6.0–8.0	59<br>59	1.005<br>5	1.025<br>8	1.015<br>6	1.010–1.020<br>5–7
hs–CRP (mg/L)	Low: <10<br>Average: 10–30<br>High: >30	174	0.1	320.8	5.2	1.2–19.7

### Biochemical and hematological analysis of COVID-19 positive and negative subjects

Out of the 174 subjects in the study, 121 were COVID-19 positive, and 53 were COVID-19 negative. Our results demonstrated that median CRP was significantly higher in the infected group compared to the uninfected group (Table 2). Regarding kidney function parameters, median creatinine concentration was significantly higher in COVID-19 patients, whereas median sodium was significantly lower in this group. Additionally, the COVID-19 positive group had a significantly lower median eGFR of 86.6 mL/min/1.73 m^2^ compared to the negative group. Other measured biochemical parameters did not show any significant changes. The median WBC count was significantly lower in COVID-19 positive subjects compared to their negative counterparts.

**Table 2:** Biochemical parameters in COVID-19 positive and negative subjects No significance testing was done for basophils since almost all COVID-19 positive and negative subjects had 0 basophils.

Variable	COVID-19 positive	COVID-19 negative	p-value
Age (years)	50 (30.5–67.5)<br>N=121	49 (38–60.5)<br>N=53	0.756
BMI (kg/m^2^)	26.2 (24.6–29.7)<br>N=64	27.8 (24.2–31.0)<br>N=52	0.615
Kidney function<br><br>Creatinine (μmol/L<br>Uric acid (mmol/L)<br>Potassium (mmol/L)<br>Sodium (mmol/L)<br>Chloride (mmol/L)<br>eGFR (mL/min/1.73 m^2^<br> BUN (mmol/L<br> BUN/creatinine	N=121<br><br>79.5 (61.8–114.9)<br>0.31 (0.23–0.38)<br>4.3 (4.1–4.6)<br>135 (132–139)<br>102 (100–105)<br>86.6 (56.8–112.2)<br>5.17(3.7–9.6)<br>18 (13.4–23.3)	N=53<br><br>70.7 (61.8–88.4)<br>0.33 (0.28–0.42)<br>4.2 (4.0–4.5)<br>138 (135–139)<br>103 (102–105.5)<br>98.8 (76.5–110.8)<br>4.8 (4.0–6.4)<br>16.55 (13.5–21.9)	<br>0.018<br>0.051<br>0.300<br><0.0001<br>0.066<br>0.032<br>0.1230.400
CBC<br>Hb (g/L)<br>Hct (%)<br>RBC (10^12^/L)<br>MCV (fL)<br>MCH (pg)<br>MCHC (g/L)<br>RDW (%)<br>MPV (fL)<br>WBC (10^9^/L)<br>Neutrophils (%)<br>Lymphocytes (%)<br>Monocytes (%)<br>Eosinophils (%)<br>Basophils (%)<br>Platelets (10^9^/L)<br>Neutrophil/lymphocyte	N=46<br>141 (134–158)<br>43.1 (±6.6)<br>4.9 (±.8)<br>88.5 (85–93)<br>29.0 (28–31)<br>331 (324–337)<br>13.7 (13.3–14.2)<br>9.2 (8.7–9.6)<br>6.3 (4.9–7.6)<br>57.6 (±16.7)<br>33.0 (±15.2)<br>8 (6–10)<br>1 (1–2)<br>0.02 (±.15)<br>245.5 (195.8–317)<br>1.7 (1.2–3.1)	N=49<br>147 (135–165)<br>44.0 (±5.4)<br>5.0 (±.6)<br>89.4 (86.7–93.4)<br>30.1 (29.1–31.6)<br>336 (332–342)<br>14.0 (13.5–14.9)<br>8.4 (8.0–9.0)<br>7.5 (5.9–9.3)<br>58.2 (±10.3)<br>31.3 (±10.0)<br>8 (6–9)<br>2 (1–3)<br>0.04 (±.20)<br>237 (210.5–289.5)<br>1.9 (1.3–2.7)<br>	<br>0.197<br>0.426<br>0.580<br>0.685<br>0.009<br><0.0001<br>0.026<br><0.0001<br>0.015<br>0.830<br>0.511<br>0.609<br>0.016<br>-<br>0.944<br>0.559
Urinalysis<br>Urine specific gravity	N=34<br>1.0100 (1.0100–1.0163)	N=25<br>1.0150 (1.0100–1.0200)	0.083
Urine chemical and microscopic<br>examination<br><br>Colour (Yellow – brown)<br>Transparency (Not clear)<br>Nitrite (positive)<br>Ketone (positive)<br>Protein (positive)<br>Glucose (positive)<br>Bilirubin<br>WBC (positive)<br>RBC (positive)<br>Epithelial cells (positive)<br>Mucus (positive)<br>Casts (positive)<br>Crystals (positive)<br>Parasites<br>Bacteria (positive)<br>Urine pH	N=34<br><br> 1 (2.9%)<br>32 (94.1%)<br>2 (5.9%)<br>1 (2.9%)<br>18 (52.9%)<br>13 (38.2%)<br>All negative<br>8 (23.5%)<br>8 (23.5%)<br>23 (67.6%)<br>23 (67.6%)<br>1 (2.9%)<br>1 (2.9%)<br>All negative<br>1 (2.9%)<br>7 (5–7)	N=25<br><br>0 (0.0%)<br> 24 (96%)<br> 1 (4.0%)<br>0 (0.0%)<br>3 (12.0%)<br>4 (16.0%)<br>All negative<br>4 (16.0%)<br>8 (32.0%)<br>19 (76.0%)<br>19 (76.0%)<br>0 (0.0%)<br>0 (0.0%)<br>All negative<br>0 (0.0%)<br>6 (5–6)	<br><br>1.000<br>1.000<br>1.000<br>1.000<br>0.001<br>0.062<br>-<br>0.478<br>0.470<br>0.484<br>0.484<br>1.000<br>1.000<br>-<br>1.000<br>0.020
hs-CRP (mg/L)	N=1219.8 (4.74–32.39)	N=530.8 (0.60–1.40)	<0.0001

Additionally, MCH, MCHC, RDW, and eosinophils were significantly lower in the infected group compared to the non-infected subjects, while MPV level was higher in the COVID-19 group compared to non-infected subjects (Table 2). Urine analysis indicated that COVID-19 patients had a significantly higher median urine pH than uninfected subjects. Proteinuria was positive in 53% of COVID-19 patients compared to only 12% positive in the uninfected group. No significant difference in all other parameters was examined in urine (Table 2). In our study, there were no significant differences between COVID-19 infected and uninfected groups in gender, smoking, presence of chronic diseases, or type of medications used (Table 3). In addition, our analyzed data revealed that COVID-19 positive subjects in the present study suffered mainly from fever (95%) and fatigue (92%), whereas vomiting (28%) and diarrhea (38%) were the least common (Table 4). Finally, the most commonly used food supplement by COVID-19 patients was vitamin C (58.7%), followed by Zinc supplements (54.5%).

**Table 3:** Demographic parameters in COVID-19 positive and negative subjects The p-value represents the result of Chi-square test or Fisher’s exact test.<br>^1^ Diabetes medication includes: Galvs, Glucophage and Janumet,<br>^2^ Hypertension medication includes: Blopress, Concor, Nebilet, Diovan.<br>^3^ Hyperlipidemia medication includes: Crestor, Lipitor, Liptrol, and Lovastatin.

Variable	COVID-19 positive<br>N=121	COVID-19 negative<br>N=53	p-value
Male	64 (52.9%)	26 (49.1%)	0.641
Smoking	29 (24%)	12 (22.6%)	0.850
Chronic diseases<br>Hyperlipidemia<br> Diabetes<br> Hypertension<br> Hypothyroidism<br> Leukemia<br> Overall chronic disease patients	N=121<br>10 (8.3%)<br>32 (26.4%)<br>33 (27.3%)<br>1 (0.8%)<br>0 (0.0%)<br>67 (55.4%)	N=53<br>7 (13.2%)<br>12 (22.6%)<br>15 (28.3%)<br>2 (3.8%)<br>1 (1.9%)<br>22 (41.5%)	<br> 0.312<br>0.595<br>0.889<br>0.220<br>0.305<br>0.092
Patient taking medication<br>Diabetes medication^1^<br> Hypertension medication^2^<br>Hyperlipidemia medication^3^	52 (43%)<br>30 (24.8%)<br>32 (26.4%)<br>11 (9.1%)<br>	31 (58.5%)<br>12 (22.6%)<br>14 (26.4%)<br>7 (13.2%)<br>	0.059<br>0.760<br>0.997<br>0.412

**Table 4:** Frequencies for COVID-19 symptoms and home remedies used in COVID-19 positive patients

	Frequency (%)
COVID-19 symptoms<br>Fever<br>Cough<br>Difficulty breathing<br>Headache<br>Difficulty concentrating<br>Chest pain<br>Vomiting<br>Diarrhoea<br>Loss of taste and smell<br>Muscle soreness<br>Fatigue<br>Home remedy treatments used for COVID-19 infection<br>Anise<br>Antibiotics<br>Antivirals<br>Aspirin<br>Chamomile<br>Ginger<br>Herbs<br>Lemon<br>Panadol<br>Viramin C<br>Viramin D<br>Zinc<br>No treatments used	N=121<br>115 (95.0%)<br>89 (73.6%)<br>53 (43.8%)<br>75 (62.0%)<br>67 (55.4%)<br>57 (47.1%)<br>34 (28.1%)<br>46 (38.0%)<br>55 (45.5%)<br>61 (50.4%)<br>111 (91.7%)<br><br>2 (1.7%)<br>24 (19.8%)<br>3 (2.5%)<br>9 (7.4%)<br>1 (0.8%)<br>1 (0.8%)<br>6 (5.0%)<br>5 (4.1%)<br>55 (45.5%)<br>71 (58.7%)<br>53 (43.8%)<br>66 (54.5%)<br>5 (4.1%)

### Biochemical and hematological analysis of severe and non-severe COVID-19 positive subjects

To study the relationship between the severity of COVID-19 infection and the markers of kidney injury, COVID-19 patients were allocated into two groups based on CRP values. Those who had CRP ≥33.55 mg/L were classified as having a severe illness, and those with CRP levels lower than this cut-off value as having a non-severe illness. This classification was according to Hariyanto et al. [15]. Accordingly, 29 patients were found to have severe COVID-19 illness at the time of sample collection, while 92 patients had a non-severe illness (Table 5). The severe group had a median CRP of 73 mg/L, whereas the non-severe group had a median of 6.3 mg/L. Our data demonstrated that patients in the severe illness group were significantly older, had a significantly higher median BMI, and their symptoms lasted longer than the non-severe group. Moreover, the following kidney parameters were significantly higher in the severe groups: creatinine, BUN, and BUN/creatinine ratio, while sodium and eGFR were significantly lower in the severe group. The majority (57.6%) of the non-severe illness group had an eGFR of 90 mL/min/1.73 m^2^ or higher compared to only 3.4% in the severe group. Apparently, the severe COVID-19 group had significantly lower hemoglobin, hematocrit, RBC count, lymphocytes percent, and platelet count and higher MPV value, neutrophil percent, and a neutrophil/Lymphocyte ratio compared to the non-severe group. Our results clarified that a higher percentage of severe COVID-19 infection group had diabetes, hypertension, and chronic diseases in general. Consequently, the severe COVID-19 infection group had a higher percentage of members taking medications for chronic diseases (Table 6).

**Table 5:** Biochemical parameters in COVID-19 positive samples categorized based on the severity of infection *For the significance testing for levels of eGFR, a post-hoc residual test was used, and a multiple testing correction was applied making the significance level 0.01 instead of 0.05.

Variable	Severe group<br>COVID-19 positive<br>(CRP≥33.55 mg/L)	Non-severe group<br>COVID-19 positive<br>(CRP<33.55 mg/L)	p-value
hs-CRP (mg/L)	73.0 (54.9–111.9)<br>N=29	6.3 (3.9–15.2)<br>N=92	
Age (year)	69 (56.5–80.5)<br>N=29	39 (29–59.5)<br>N=92	<0.0001
BMI (kg/m^2^)	28.8 (26.7–33.4)<br>N=12	26.0 (24.4–29.5)<br>N=52	0.01
Duration from symptom onset<br>(days)	5 (3.5–6)<br>N=29	3 (2–5)<br>N=92	0.004
Kidney function<br>Creatinine, μmol/L<br>Uric acid, mmol/L<br>Potassium, mmol/L<br>Sodium, mmol/L<br>Chloride, mmol/L<br>eGFR, mL/min/1.73 m^2^<br>BUN, mmol/L<br>BUN/creatinine	N=29<br><br> 114.9 (97.2–167.9)<br> 0.31 (0.23–0.46)<br> 4.4±0.5<br> 132.0 (131.5–135.0)<br> 102.0 (99.0–105.5)<br> 48.5 ± 21.1<br> 10.2 (9.3–20.3)	N=92<br><br> 70.7 (61.8–97.2)<br> 0.31 (0.23–0.37)<br> 4.3±0.4<br> 135.0 (133.3–139.0)<br>102.0 (100.0–104.8)<br> 93.9 ± 31.3<br> 4.6 (3.6–6.1)<br>	<br><0.0001<br> 0.327<br> 0.321<br> 0.005<br> 0.774<br> <0.0001<br> <0.0001<br> <0.0001
CBC<br>Hb (g/L)<br> Hct (%)<br> RBC (10^12^/L)<br>MCV (fL)<br>MCH (pg)<br>MCHC (g/L)<br>RDW (%)<br>MPV (fL)<br>WBC (10^9^/L)<br>Neutrophils (%)<br>Lymphocytes (%)<br>Monocytes (%)<br>Eosinophils (%)<br>Basophils (%)<br>Platelets (10^9^/L)<br>Neutrophil/lymphocyte ratio	N=6<br>103 (94–122)<br>31.4 (29.4–36.7)<br>4.1 ± 1.1<br>88.5 (76.5–91.8)<br>28.0 (25.0–31.3)<br>329 (319–341)<br>14.3 (13.5–17.8)<br>10.6 (9.2–11.1)<br>7.1 (6.0–12.8)<br>74.0 ± 13.3<br>18.8 ± 13.0<br>5.8 ± 3.1<br>1.0 (0.8–2.0)<br>0.0 (0.0–0.3)<br>178.7 ± 57.1<br>3.3 (2.0–27.7)	N=40<br>146 (135–158)<br>45.1 (41.1–48.1)<br>5.0 ± 0.7<br>88.5 (85.0–93.0)<br>29.0 (28.0–30.9)<br>331 (324–338)<br>13.6 (13.3–14.1)<br>9.2 (8.6–9.6)<br>5.9 (4.8–7.3)<br>55.1 ± 15.9<br>35.2 ± 14.5<br>8.2 ± 2.9<br>1.0 (1.0–2.0)<br>0.0 (0.0–0.0)<br>263.7 ± 87.<br>1.5 (1.1–2.4)	<br>0.001<br><0.0001<br>0.015<br>0.659<br>0.465<br>0.769<br>0.137<br>0.023<br>0.134<br>0.008<br>0.013<br>0.079<br>0.423<br>–<br>0.026<br>0.01
Urinalysis<br>Urine specific gravity<br>Urine pH<br>Transparency of urine<br>(Not clear)<br>Urine protein (Positive)<br>Urine glucose (Positive)<br>Urine WBC (Positive)<br>Urine RBC (Positive)<br>Urine epithelial cells (Positive)<br>Urine mucus (Positive)<br>Levels of eGFR<br>eGFR ≥90<br>60≤ eGFR <90<br>30≤ eGFR <60<br>15≤ eGFR <30<br>eGFR <15	N=10<br> 1.010 (1.005–1.010)<br> 7 (7–8)<br> 10 (100.0%)<br> 9 (90.0%)<br> 7 (70.0%)<br> 1 (10.0%)<br> 3 (30.0%)<br> 7 (70.0%)<br> 7 (70.0%)<br> N=29<br> 1 (3.4%)<br> 6 (20.7%)<br> 15 (51.7%)<br> 6 (20.7%)<br> 1 (3.4%)	N=24<br> 1.015 (1.010–1.020)<br> 6 (5–7)<br> 22 (91.7%)<br> 9 (37.5%)<br> 6 (25.0%)<br> 7 (29.2%)<br> 5 (20.8%)<br> 16 (66.7%)<br> 16 (66.7%)<br> N=92<br> 53 (57.6%)<br> 25 (27.2%)<br> 10 (10.9%)<br> 4 (4.3%)<br> 0 (0.0%)	<br>0.006<br>0.026<br>1.000<br>0.008<br>0.022<br>0.385<br>0.666<br>1.000<br>1.000<br><br><0.0001*<br>0.484<br><0.0001*<br>0.005*<br>0.072

**Table 6:** Frequencies of demographic parameters in COVID-19 positive samples categorized based on the severity of infection The p-value represents the result of the Chi-square test or Fisher’s exact test

Variable	Severe group<br>(CRP≥33.55 mg/L)	Non-severe group<br>(CRP<33.55 mg/L)	p-value
Gender (Male)	N=29<br>14 (48.3%)	N=92<br>43 (46.7%)	0.885
Smoking	4 (13.8%)	25 (27.2%)	0.141
Chronic diseases<br>Hyperlipidemia<br>Diabetes<br>Hypertension<br>Chronic diseases	<br>1 (3.4%)<br>19 (65.5%)<br>15 (51.7%)<br>26 (89.7%)	<br>9 (9.8%)<br>13 (14.1%)<br>18 (19.6%)<br>28 (30.4%)	<br>0.280<br><0.0001<br>0.001<br><0.0001
Taking medications (ARBS and<br> ACE inhibitors, etc.)	26 (89.7%)	26 (28.3%)	<0.0001
Taking supplements (Vit C, D,<br> Zinc, etc.)	21 (72.4%)	74 (80.4%)	0.359
Regular consumption of certain<br> foods (Broccoli, honey, ginger,<br> garlic, onion, herbs, etc.)	25 (86.2%)	68 (73.9%)	0.171
Used home remedies for<br> COVID-19 infection(Anise, ginger, lemon, etc.)	29 (100.0%)	87 (94.6%)	0.336
COVID-19 symptoms<br>Fever<br>Cough<br>Difficulty breathing<br>Headache<br>Difficulty concentrating<br>Chest pain<br>Vomiting<br>Diarrhea<br>Loss of taste and smell<br>Muscle soreness<br>Fatigue	<br>27 (93.1%)<br>21 (72.4%)<br>15 (51.7%)<br>18 (62.1%)<br>18 (62.1%)<br>16 (55.2%)<br>10 (34.5%)<br>13 (44.8%)<br>11 (37.9%)<br>16 (55.2%)<br>26 (89.7%)	<br>88 (95.7%)<br>68 (73.9%)<br>38 (41.3%)<br>57 (62.0%)<br>49 (53.3%)<br>41 (44.6%)<br>24 (26.1%)<br>33 (35.9%)<br>44 (47.8%)<br>45 (48.9%)<br>85 (92.4%)	<br>0.629<br>0.873<br>0.324<br>0.991<br>0.405<br>0.318<br>0.380<br>0.386<br>0.351<br>0.557<br>0.641

## Comparing COVID-19 patients with high creatinine and normal creatinine levels

To study the characteristics of COVID-19 patients with elevated creatinine levels (>123.7 μmol/L), patients were divided based on this cut-off value, and all clinical parameters were compared. Accordingly, 18 patients (14.87%) were found to have elevated creatinine levels (>123.7 μmol/L), while 103 had creatinine levels below 123.7 μmol/L. As shown in Table 7, COVID-19 patients with elevated creatinine had a significantly higher median CRP, older age, and were symptomatic for a longer period than patients with normal creatinine. Moreover, BUN and BUN/creatinine ratios were significantly higher in the elevated creatinine group, whereas eGFR was significantly lower. About half the patients with elevated creatinine had an eGFR between 15 and 30 mL/min/1.73 m^2^, whereas none of the patients in the normal group had eGFR at this level. In the same context, the group of patients with elevated creatinine levels had a significantly higher percentage of subjects with diabetes, hypertension, and chronic diseases in general and a higher percentage of subjects taking medications for chronic illness. Finally, the effect of risk factors contributing to elevated creatinine levels above 123.7 μmol/L for the COVID-19 positive group was investigated using binary logistic regression analysis. *Table VIII* indicates that taking medications for chronic diseases was the risk factor with the highest odds ratio for being in the elevated creatinine group, making these patients 15.3 times more likely to be in the elevated creatinine group relative to patients who do not take medications. Both hypertension and diabetes were found to increase the likelihood of being in the elevated creatinine group by 8.2 and 3.4 times, respectively, relative to patients without hypertension and diabetes. Moreover, patients who suffered from a severe infection, as determined by having a CRP value higher than 33.55 mg/L, were 7.3 times more likely to have elevated creatinine than patients in the non-severe COVID-19 infection group. Finally, each additional day since the onset of symptoms increased the likelihood of having elevated creatinine by 1.7 times (*Table VIII*).

**Table 7:** Biochemical and frequencies of demographic parameters in COVID-19 positive Samples categorized based on creatinine concentration *For the significance testing for levels of eGFR, a post-hoc residual test was used, and a multiple testing correction was applied making the significance level 0.01 instead of 0.05.

Variable	Elevated creatinine<br>COVID-19 positive<br>(>123.7 μmol/L)	Normal creatinine<br>COVID-19 positive<br>(≤123.7 μmol/L)	p-value
hs-CRP (mg/L)	49.1 (23.7–144.6)<br>N=18	7.5 (4.2–21.2)<br>N=103	<0.0001
Age (years)	62 (50–78.5)<br>N=18	45 (30–64)<br>N=103	0.004
BMI (kg/m^2^)	27.7 (24.6–38.3)<br>N=5	26.2 (24.5–29.7)<br>N=59	0.381
Duration from onset of symptoms<br> (days)	6 (4–7)<br>N=18	3 (2–5)<br>N=103	0.001
Kidney function<br><br>Creatinine (μmol/L)<br>Sodium (mmol/L)<br>eGFR (mL/min/1.73 m^2^)<br>BUN mmol/L	N=18<br>167.9 (141.4–203.3)<br>134 (131.8–140.3)<br>27.1 (23.4–38.9)<br>17.5 (11.1–43.0)<br>25.2 (17–34.4)	N=103<br>70.7(60.1– 97.2)<br>135 (132–139)<br>93.3 (67.7–115.9)<br>4.8 (3.6–7.5)<br>17.3 (12.7–22.2)	<br>–<br>0.575<br><0.0001<br><0.0001<br>0.002
CBC<br>Hb (g/L)<br>Hct (%)<br>RBC (10^12^/L)<br>Neutrophils (%)<br>Lymphocytes (%)<br>Platelets (10^9^/L)<br>Neutrophil/lymphocyte	N=5<br>95 (85–126)<br>29.7 (27.6–37.7)<br>3.4 (2.9–4)<br>85 (63–94.5)<br>4 (2.5–30.5)<br>130 (102–225)<br>21.3 (2.3–39.3)	N=41<br>144 (135–158)<br>44.8 (40.6–48)<br>5.1 (4.5–5.5)<br>55 (48.5–63.5)<br>33 (27–42)<br>254 (209.5–323)<br>1.7 (1.2–2.3)	<br>0.004<br>0.004<br>0.001<br>0.009<br>0.012<br>0.012<br>0.012
Urinalysis<br>Urine specific gravity<br>Urine pH<br>Urine protein (positive)<br>Urine glucose (positive)<br>Urine WBC (positive)<br>Urine RBC (positive)<br>Levels of eGFR<br>eGFR ≥90<br>60 eGFR <90<br>30 eGFR <60<br>15 eGFR <30<br>eGFR <15	N=8<br>1.0075 (1.005–1.010)<br>7 (7–7.75)<br>8 (100%)<br>8 (100%)<br>0 (0%)<br>0 (0%)<br>N=18<br>0 (0%)<br>1 (5.6%)<br>6 (33.3%)<br>10 (55.6%)<br>1 (5.6%)	N=26<br>1.0150 (1.010–1.020)<br>6 (5–7)<br>10 (38.5%)<br>5 (19.2%)<br>8 (30.8%)<br>8 (30.8%)<br>N=103<br>54 (52.4%)<br>30 (29.1%)<br>19 (18.4%)<br>0 (0%)<br>0 (0%)	<br>0.006<br>0.020<br>0.003<br><0.0001<br>0.152<br>0.152<br><br><0.0001*<br>0.035<br>0.150<br><0.0001*<br>0.016
Gender (male)	9 (50%)	48 (46.6%)	0.790
Chronic diseases<br>Hyperlipidemia<br>Diabetes<br>Hypertension<br>Chronic diseases<br>Taking medications	<br>2 (11.1%)<br>9 (50%)<br>12 (66.7%)<br>16 (88.9%)<br>16 (88.9%)	<br>8 (7.8%)<br>23 (22.3%)<br>21 (20.4%)<br>38 (36.9%)<br>36 (35%)	<br>0.634<br>0.021<br><0.0001<br><0.0001<br><0.0001

## Discussion

To our knowledge, this is the first study in Jordan conducted to investigate the influence of COVID-19 infection on kidney functionality and to correlate the severity of disease to various serum biochemical and hematological parameters in addition to urine analysis in infected patients and healthy volunteers. CRP is a sensitive biomarker for inflammation. The increase in CRP may result from the SARS-CoV-2 virus or bacterial co-infection in severe COVID-19 cases [15]. In the present investigation, CRP was higher in COVID-19 patients compared to the COVID-19 negative group. A study revealed a significant increase in CRP level in COVID-19 patients with an average of 20 to 50 mg/L, which further increases with disease severity [16]. The severe COVID-19 group in our study has a significant increase in CRP with a median range of 73 mg/L compared to the non-severe group which had a median of 6.3 mg/L. Regarding kidney function parameters, our results demonstrated that the median creatinine concentration was significantly higher in COVID-19 patients when compared to the COVID-19 negative group. Moreover, the median creatinine level was significantly higher in the severe COVID-19 group than in the non-severe one. Cheng et al. [17] reported that an elevated serum creatinine level upon admission was associated with an increase in the probability of patients being admitted to the intensive care unit and using mechanical ventilation; thus, kidney disease on admission exhibits an increased risk of deterioration. Our data demonstrated that blood urea, BUN level, and BUN/creatinine ratio were significantly higher in the severe group than in the non-severe group. Researchers found that inflammation and cytokine storms that were induced by COVID-19 infection, particularly in severe cases, can cause a significant increase in blood urea reabsorption and hence, increase the BUN concentration and BUN/creatinine ratio [18]. Noteworthy, BUN/creatinine ratio was elevated in COVID-19 patients who developed AKI after hospital admission [19]. The present study demonstrated that the positive COVID-19 group had a significantly lower eGFR which further decreased significantly with disease severity. In fact, 55.6% of the high creatinine group have eGFR between 15 and 30 mL/min/1.73 m^2^. It has been found that a high mortality rate among COVID-19 patients and a higher probability of developing AKI were associated with lower GFR (51-85 mL/min/1.73 m^2^) [20] [21]. Concerning electrolytes levels, our results demonstrated that serum sodium concentration was significantly lower in COVID-19 patients and even lower in the severe COVID-19 positive group compared to the non-severe group. Hyponatremia is the most prevalent electrolyte disorder in COVID-19 patients, and it is mainly induced due to inappropriate secretion of antidiuretic hormone [22] [23]. Regarding urine analysis, the protein was detected in over half of COVID-19 patients, while 90% of the severe COVID-19 group had a positive result for urine protein compared with 37.5% of the non-sever group. Clearly, proteinuria is common in COVID-19 patients, indicating tubular damage as the infiltration of protein in the kidney is compromised, and reabsorption does not occur appropriately [20]. Moreover, our findings revealed that COVID-19 patients had a significantly higher median urine pH than uninfected subjects in the severely ill COVID-19 patients and in the high creatinine group. Additionally, our data demonstrated that the urine-specific gravity significantly decreased in severe cases of COVID-19 infection compared to non-severe cases. These findings are consistent with studies that confirmed that COVID-19 patients had a higher urine pH level and lowered specific gravity than the control group [24]. Considering hematological alterations in COVID-19 patients, our results showed a lower hemoglobin level in the severe and high creatinine groups. Bergamaschi et al. [25] confirmed the prevalence of anemia among COVID-19 patients, which was 61% compared to 45% in a control group. In the same context, our data revealed that COVID-19 patients had a lower MCH, MCHC, and RDW. A study declared that lower MCV and MCH were significantly associated with disease severity, which may be due to oxidative stress that increases significantly with inflammation and triggers the release of oxygen free radicals [26]. Our results demonstrated that the WBC count was higher in the severe group, but the difference was insignificant. In the same context, our data revealed that a significantly lower lymphocyte percentage accompanied the severity of illness while neutrophils and neutrophil/lymphocyte ratio increased significantly in the severe group. It has been reported that COVID-19 patients are presented with increased WBC and neutrophil counts, while lymphopenia and eosinopenia were observed in all positive groups [27]. Platelets showed a significant decrease in count and increase in MPV accompanied by disease severity. This decrease may be due to the direct effect of infection on bone marrow function and its correlated cytokines storm, which causes destruction in bone marrow progenitor cells and inhibition of platelet synthesis [28]. MPV is considered an important indicator of both proinflammatory and prothrombotic conditions that is influenced by IL-1, IL-6, and other related cytokines, which regulate thrombopoiesis and cause the production of new immature platelets by the side of platelets destruction [29] [30]. The elucidation of risk factors leading to kidney impairment in COVID-19 infection is important for physicians to manage their patients better. Our data revealed that patients in the severe COVID-19 illness group were significantly older, had a higher BMI, and their symptoms had lasted longer than the non-severe group. Older age was previously reported to be associated with AKI [31]. Furthermore, the correlation of obesity with COVID-19 infection was confirmed in several studies, which found that obese people have a significantly higher probability of developing CKD or AKI [32]. However, our study illustrated no gender differences in disease severity. Interestingly, a recent study in Jordan indicated that females were more prone to COVID-19 infection than males [33]. Finally, our study suggested a strong association between taking medications for chronic diseases and elevated creatinine levels in COVID-19 patients. In fact, many therapeutic agents show some degree of nephrotoxicity, leading to lower GFR [34]. Moreover, our data indicated that hypertension was the most common comorbidity among COVID-19 patients who exhibited elevated creatinine levels, followed by diabetes. It has been reported that the most frequent causes of AKI in COVID-19 patients were prerenal and pre-existent hypertension [35]. In addition, diabetic patients were the most susceptible to severe disease and hospitalization as these patients suffer from chronic hyperglycemic and continuous inflammation [36]. Finally, a study revealed that all these comorbidities increase the risk of developing AKI and thus increase the mortality rate and risk at any time of hospitalization [35].

## Conclusion

Our study demonstrated that COVID-19 infection affects kidney function. Moreover, the severity of the disease is correlated with many biochemical markers of kidney injury. Comorbidities such as older age (≥65), hypertension, diabetes, taking medications, and the severity of the disease are risk factors that are more likely to contribute to kidney impairment in COVID-19 positive patients. In our sample, the prevalence of kidney impairment in COVID-19 patients based on elevated creatinine levels was 13.87%. Therefore, clinicians should be aware of kidney disease in patients with severe COVID-19, given that a high prevalence of under-diagnosed CKD has been reported among Jordanians [12]. Finally, Comorbidities should be monitored closely in COVID-19 patients to minimize the risk of progression to severe disease and kidney impairment.

## Dodatak

### Conflict of interest statement

All the authors declare that they have no conflict of interest in this work.

### List of abbreviations

BMI, body mass index;<br>eGFR, estimated glomerular filtration rate;<br>BUN, blood urea nitrogen;<br>CBC,complete blood count;<br>Hb, hemoglobin;<br>Hct, hematocrit;<br>RBC, red blood cell;<br>MCV, mean corpuscular volume;<br>MCH, mean corpuscular hemoglobin;<br>MCHC, mean corpuscular hemoglobin concentration;<br>RDW, red cell distribution width;<br>MPV, mean platelet volume;<br>WBC, white blood cell;<br>CRP, c-reactive protein;<br>N, number of subjects with available measurements;<br>CI, confidence interval for the odds ratio.
